# Retinal and Choroidal Structural and Microvascular Characteristics in Women With Polycystic Ovary Syndrome: A Systematic Review and Meta-Analysis of Optical Coherence Tomography (OCT) and OCT Angiography (OCTA) Studies

**DOI:** 10.7759/cureus.104504

**Published:** 2026-03-01

**Authors:** Abdullah Bousamri, Mohammad Al-Shehri, Mohammad Kana'an, Talal Almutairi, Faisal Alharbi, Ahmad Aburezq, Abdulaziz Bousamri

**Affiliations:** 1 Ophthalmology, Faculty of Medicine, Jordan University of Science and Technology, Irbid, JOR; 2 General Practice, Farwaniya Hospital, Kuwait City, KWT; 3 Ophthalmology, Faculty of Medicine, Kuwait University, Kuwait City, KWT

**Keywords:** choroid, optical coherence tomography, optical coherence tomography angiography, polycystic ovary syndrome, retina

## Abstract

Polycystic ovary syndrome (PCOS) is a complex endocrine disorder that involves metabolic, hormonal, and inflammatory changes. These changes can affect tissues with high metabolic needs and sensitive microvascular systems. Optical coherence tomography (OCT) and OCT angiography (OCTA) are non-invasive ways to assess the structure and blood vessels of the retina and choroid. However, studies on women with PCOS have shown mixed results. This systematic review and meta-analysis set out to combine and analyze the available evidence on how PCOS affects retinal structure and microvasculature. We searched PubMed, Embase, Scopus, Web of Science, Cochrane CENTRAL, and Google Scholar from inception to February 1, 2026, for observational studies comparing OCT and OCTA results in women with PCOS and healthy controls. The main outcomes were average peripapillary retinal nerve fiber layer (RNFL) thickness, central macular thickness (CMT), subfoveal choroidal thickness (SFCT), and vessel density in the superficial and deep capillary plexus (SCP, DCP). We used the Newcastle-Ottawa Scale (NOS) to check study quality and the GRADE framework to rate the certainty of the evidence. We performed random-effects meta-analyses using mean differences (MDs) and 95% confidence intervals (CIs). We included 15 observational studies with a total of 1,370 women (737 with PCOS and 633 controls), and 13 studies were used in the quantitative analysis. Women with PCOS had a higher average RNFL thickness (MD +3.75 µm; 95% CI 2.48 to 5.01; moderate-certainty evidence) and a small but significant decrease in CMT (MD −3.29 µm; 95% CI −6.46 to −0.11; moderate certainty) compared to controls. SFCT was also higher overall (MD +58.03 µm), but there was a lot of variation between studies (*I²* = 99%). Subgroup analysis showed that SFCT thickening was only seen in studies where disease duration was not specified, while studies with newly diagnosed PCOS patients found no significant difference. There were no significant differences in SCP or DCP vessel density (low-certainty evidence).​ PCOS is linked to consistent changes in the retina and choroid, including thicker RNFL and thinner central macula, while the density of retinal blood vessels seems unchanged. Choroidal thickening was only found in groups where the length of disease was not specified, whereas the newly diagnosed showed no change. This shows that future studies should track disease duration over time to better understand these changes.

## Introduction and background

Polycystic ovary syndrome (PCOS) is the most common endocrine condition in women of reproductive age, with a global prevalence of 8-13% [1]. Although the syndrome is classically defined as a reproductive dysfunction, it is now established to be a multi-system metabolic disorder characterized by hyperandrogenism, insulin resistance, chronic low-grade inflammation, and endothelial dysfunction [1-4]. These processes are pathophysiologically interrelated and could impose greater cardiometabolic risk and have the potential to impact organs with high metabolic demand and highly regulated microvascular structures in this affected population [1,3,5,6]. To this end, the recognition of sensitive biomarkers that can be used to assess early systemic involvement in PCOS remains a gap yet to be filled in both clinical and research practice. Identifying early structural or microvascular alterations may help clarify whether PCOS exerts subclinical end-organ effects beyond the reproductive system and may provide insight into systemic disease burden.

The retina and choroid are highly sensitive neurovascular tissues that are susceptible to systemic metabolic and inflammatory changes. Retinal neurons, glial cells, and the microvasculature function as a combined unit, such that modest disturbances in perfusion, axonal transport, or inflammatory signaling may result in measurable structural alterations [7,8]. The choroid, which is among the most highly vascularized tissues in the human body, is particularly sensitive to hormonal, autonomic, and inflammatory effects [6]. Optical coherence tomography (OCT) and OCT angiography (OCTA) are both non-invasive, in vivo imaging modalities that permit high spatial-resolution imaging of retinal layer thickness, choroidal architecture, and retinal microvasculature, making both highly suitable for identifying subclinical ocular involvement in systemic disease [9].

Over the last 15 years, observational studies have evaluated OCT and OCTA in women with PCOS. Structural findings that have been reported include peripapillary retinal nerve fiber layer (RNFL) thickness changes, changes in macular thickness measures, and an increase in subfoveal choroidal thickness (SFCT), but OCTA-based measurements of retinal microvascular density have not been consistent [10-12]. The limitations of the available literature include small sample size, differences in imaging platform and acquisition protocols, variation in diagnostic criteria, and missing reporting of clinically relevant modifiers such as body mass index (BMI) and the duration of onset of disease.  As a result, the extent and clinical significance of ocular structural and microvascular changes associated with PCOS remain uncertain. 

To date, this evidence has been synthesized in a limited number of qualitative or integrative reviews, which summarized OCT and OCTA findings without providing quantitative pooled estimates [13,14]. Consequently, it remains unclear whether the reported retinal and choroidal structural changes represent reproducible characteristics of PCOS or are independent of microvascular alterations. Prior reviews have also not systematically examined the influence of clinical or methodological factors on reported outcomes. As the body of primary literature has expanded, quantitative synthesis has become feasible. To our knowledge, this is the first systematic review and meta-analysis to quantitatively synthesize OCT and OCTA findings in women with PCOS while formally exploring heterogeneity according to disease duration and imaging modality.

This systematic review and meta-analysis quantitatively evaluates structural and microvascular retinal and choroidal alterations measured by OCT and OCTA in women with PCOS compared with healthy controls. The primary outcomes are average peripapillary retinal nerve fiber layer thickness, central macular thickness, subfoveal choroidal thickness, and vessel density in the superficial and deep capillary plexus. Sensitivity analyses and predefined subgroup analyses are conducted to assess heterogeneity, particularly in relation to disease duration and imaging modality.

## Review

Methods

*Protocol and Registration* 

This systematic review was conducted in accordance with the PRISMA 2020 statement and was prospectively registered in PROSPERO (CRD420251274915) [15,16]. 

Information Sources and Search Strategy

An initial methodological search was carried out on the 9th of January and then updated on the first of February 2026, in accordance with Cochrane guidelines. No language or other restrictions were imposed. Non-English full texts were translated using Google Translate, and key numerical data were cross-verified against tables and English abstracts where available. The literature search was conducted in the following databases: PubMed, Embase, Scopus, Web of Science, Cochrane CENTRAL, and Google Scholar. Using keywords relied on the population and intervention being assessed: "polycystic ovary syndrome", "optical coherence tomography", and "optical coherence tomography angiography"; full search strategies are detailed in Appendix A. For Google Scholar, we screened the first 200 results sorted by relevance.

Selection Process

After retrieval of the records, they were uploaded to EndNote X9, deduplicated, and manually screened by two authors (Ahmad A and Mohammad K) independently. After title and abstract screening, the same two authors assessed the full texts of the remaining studies. Any disagreements were resolved by discussion with Abdullah B.

Eligibility Criteria 

Comparative observational studies in which authors assessed the retinal and choroidal structural and microvascular changes via OCT and OCTA in patients with PCOS were included (1) if they established diagnostic criteria, including the Rotterdam criteria, National Institutes of Health (NIH) criteria, or Androgen Excess PCOS Society (AES) criteria; (2) if they involve the presence of an unaffected healthy control group; or (3) if they reported OCT and/or OCTA measurements in at least one of the following outcomes: peripapillary RNFL thickness, central macular thickness (CMT), SFCT, and vascular density (VD), such as superficial and deep capillary plexus (SCP, DCP).

Preprints and non-peer-reviewed studies were considered eligible if they met all predefined inclusion criteria to minimize publication bias. The exclusion criteria were studies focusing on pregnant participants or participants with ocular diseases (e.g., retinal pathology, glaucoma/optic neuropathy) or systemic conditions likely to independently affect retinal/choroidal measures (e.g., diabetes mellitus, uncontrolled hypertension). Animal studies, narrative reviews, editorials, conference abstracts without sufficient data, and studies lacking a comparator group were excluded.

Data Items

Two reviewers (Talal A and Abdulaziz B) extracted the following data from the included studies: study design, year of publication, country, mean age, total population, and sample size. Diagnosis duration, OCT/OCTA device used, BMI, and outcomes assessed.

Risk of Bias in Studies

This process was carried out by Mohammad A and Faisal A. While the Cochrane Handbook recommends ROBINS-I for non-randomized interventions, we used the Newcastle-Ottawa Scale (NOS) for quality assessment [17,18]. This decision was based on the study designs of the included literature, which were predominantly cross-sectional comparative studies rather than longitudinal intervention trials. The NOS is better suited to assess the methodological quality of observational studies by comparing distinct groups (PCOS vs. controls) at a single time point, focusing on selection definitions and comparability of the cohort. We assigned a top score of 9, scores of 7-9 were considered "good" quality, scores of 4-6 were considered "fair" quality, and scores less than 4 were considered "poor" quality.

Certainty Assessment

We used the GRADE framework to determine the overall certainty of the evidence. This method categorizes the quality of evidence as high, moderate, low, or very low, following an assessment of potential limitations such as risk of bias, inconsistency, indirectness, imprecision, and publication bias.

Data Handling and Conversions

We performed a meta-analysis on OCT and OCTA metrics, presented as mean ± standard deviation values, which were reported in no fewer than three distinct studies. Median and IQR or min-max (range) reported data were converted accordingly using Wan et al.'s equation [19].

Meta-Analytic Model

Analysis was conducted using RevMan 5.4.1, with additional analyses performed in R software (version 4.5.2). Mean differences (MDs) with 95% confidence intervals (CIs) were calculated for all OCT and OCTA outcomes. Statistical significance was set at P < 0.05. A random-effects model (DerSimonian-Laird) was applied for all analyses due to anticipated clinical and methodological diversity [20].

Heterogeneity and Sensitivity Analyses

Statistical heterogeneity was assessed using the I² statistic, with I² >50% considered indicative of substantial heterogeneity. Sensitivity analyses were performed using a leave-one-out approach, and subgroup analyses were conducted to explore potential sources of heterogeneity and assess the robustness of pooled estimates.

Publication Bias Assessment

Publication bias was assessed for outcomes, including ≥10 studies using funnel plot inspection. Egger’s regression test was performed only when ≥10 studies were available, as asymmetry tests are unreliable with smaller numbers of studies (<10). Further specific data handling is provided in Appendix B.

Results

Study Selection 

The search returned 251 records from the databases, PubMed (N = 38), Embase (N = 102), Scopus (N = 71), Web of Science (N = 35), and Cochrane CENTRAL (N = 5). After removal of 95 duplicates, 156 unique records were screened by title and abstract, of which 141 irrelevant studies were excluded. 

Fifteen studies progressed to full-text review, of which one was not retrievable. Of the remaining 14 full-text articles, two were excluded: one was a small case series without a control group (n = 1), and the other was a cohort study without ophthalmic imaging (n = 1). After full-text assessment of database-derived records, 12 studies met the inclusion criteria.

In addition, we searched Google Scholar to enhance the coverage of missed studies. We screened the first 200 records based on relevance, and it yielded three additional eligible studies. In total, 15 studies met the inclusion criteria for qualitative synthesis, and 13 studies were included in at least one quantitative meta-analysis. 

**Figure 1:**
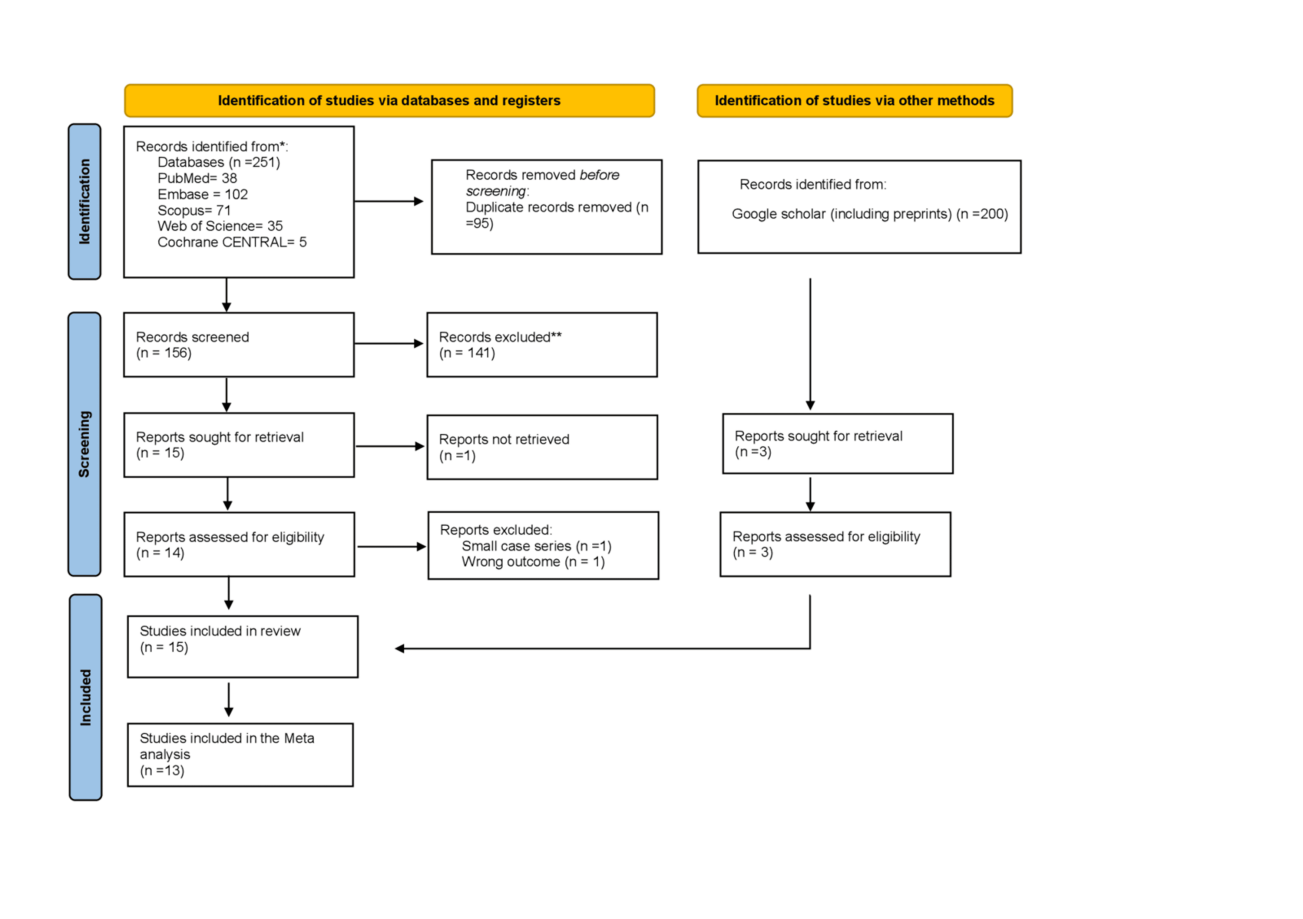
Preferred Reporting Items for Systematic Reviews and Meta-Analyses (PRISMA) flow chart of the selected studies

During quantitative synthesis, a small number of studies were excluded from specific outcome analyses due to non-extractable data, anatomical incompatibility, or biologically implausible measurements that precluded valid pooling. These exclusions were applied at the outcome level and did not necessarily apply to other outcomes reported by the same studies. Two studies, Shiromani et al. (2022), were excluded from the meta-analysis entirely because their reported outcomes did not meet the validity criteria for pooling. Sirakaya et al. (2020) did not report any of our primary outcomes. Detailed outcome-specific data handling decisions are provided in Appendix B. The study selection process is illustrated in the PRISMA flow diagram (Figure 1).

Study Characteristics 

The included studies were conducted from 2013 to 2026. They comprised a total of 1,370 women, 737 with PCOS and 633 healthy controls, with individual study sample sizes ranging from 23 to 120 participants. All studies were comparative observational designs. Most of them (N = 12) are from a single country, Turkey. The participants’ mean age ranged from 21.60 to 36.3 years across studies. Most studies diagnosed PCOS using the Rotterdam criteria, while one study applied NIH diagnostic criteria. Several studies recruited newly diagnosed PCOS patients, whereas others included participants with a longer duration of diagnosis. Six studies accounted for BMI, while only one of them had PCOS and control groups of <25 kg/m^2^. One included study was a preprint and had not undergone peer review at the time of analysis.

Regarding the OCT devices, spectral-domain systems were utilized in 12 studies to assess structural parameters. Specifically, the Heidelberg Spectralis (Heidelberg Engineering, Heidelberg, Germany) was employed in four studies. Four studies utilized Zeiss systems (Carl Zeiss Meditec, Dublin, CA, USA), including the Cirrus HD-OCT and Cirrus 5000. Additionally, two studies used the RTVue-100 (Optovue Inc., Fremont, CA, USA), and two employed Topcon 3D OCT systems (Topcon Corp., Tokyo, Japan). 

For microvascular analysis, three studies utilized the RTVue XR Avanti system (Optovue Inc., Fremont, CA, USA). Notably, all three OCTA studies employed the AngioVue software algorithm for vascular segmentation and quantification. Detailed characteristics of the included studies are summarized in Table 1.

**Table 1:** Study characteristics and participant demographics PCOS: polycystic ovary syndrome; BMI: body mass index; OCT: optical coherence tomography; OCTA: optical coherence tomography angiography; NR: not reported; RNFL: retinal nerve fiber layer thickness; CMT: central macular thickness; SFCT: subfoveal choroidal thickness; SCP: superficial capillary plexus; DCP: deep capillary plexus; FAZ: foveal avascular zone; GCC: ganglion cell complex; GCL: ganglion cell layer * Preprints (non-peer-reviewed studies)

Study	Study design	Country	Age, PCOS (years)	Age, Control (years)	Total N	PCOS N	Control N	PCOS criteria	Duration of diagnosis	OCT and OCTA device	BMI (PCOS / Control) (kg/m²)		Outcomes assessed
Acar 2021 [11]	Cross-sectional	Turkey	21.60 ± 3.39	22.21 ± 2.41	94	47	47	Rotterdam	Newly Diagnosed	Optovue RTVue XR Avanti	NR	NR	SFCT, SCP, DCP, FAZ
Acmaz 2014 [10]	Prospective controlled	Turkey	27.72 ± 5.12	35.83 ± 9.02	124	64	60	Rotterdam	NR	Heidelberg Spectralis	NR	NR	RNFL, CMT, SFCT
Adiyeke 2017 [21]	Clinical study	Turkey	27 ± 4.18	26.4 ± 3.78	100	50	50	Rotterdam	NR	SD-OCT	NR	NR	RNFL, CMT
Alpogan 2023 [22]	Cross-sectional	Turkey	25.37 ± 5.11	26.64 ± 4.96	79	34	45	Rotterdam	Newly Diagnosed	Optovue RTVue-100	23.49 ± 4.39	21.9 ± 2.6	RNFL, CMT, GCC
Demir 2013 [23]	Clinical study	Turkey	24.55 ± 5.67	27.29 ± 5.9	86	44	42	Rotterdam	NR	Optovue RTVue-100	NR	NR	RNFL, CMT, GCC
Elbeyli 2021 [12]	Cross-sectional	Turkey	36.0 ± 13.4	36.3 ± 7.7	48	23	25	Rotterdam	> 1 year	Optovue RTVue XR Avanti	26.2 ± 4.6	25.8 ± 3.8	RNFL, CMT, SCP, DCP, FAZ
Gunes^*^ 2025 [24]	Comparative observational	Turkey	22.59 ± 1.59	22.56 ± 1.57	87	41	46	Rotterdam	Newly Diagnosed	Zeiss Cirrus 5000	NR	NR	RNFL, CMT, SFCT
Icoz 2026 [25]	Case-control	Turkey	28.9 ± 9.4	27.5 ± 7.5	85	45	40	Rotterdam	Newly Diagnosed	Zeiss Cirrus HD-OCT	25.2 ± 4.7	24.8 ± 3.9	RNFL, GCC, SFCT
Junior 2015 [26]	Cross-sectional	Brazil	26.6 ± 4.7	26.8 ± 4.2	82	42	40	Rotterdam	NR	Zeiss Cirrus HD-OCT	30.6 ± 6.0	27.2 ± 4.0	RNFL, CMT
Puthiyedath 2022 [27]	Cross-sectional	India	25.73 ± 3.27	26.20 ± 3.27	60	30	30	Rotterdam	Variable (~2.5–13 Years)	Zeiss Cirrus HD-OCT	NR	NR	RNFL, CMT
Shiromani 2022 [28]	Cross-sectional	India	26.32 ± 5.84	32.09 ± 7.83	110	55	55	Rotterdam	NR	Topcon 3D SD-OCT	NR	NR	RNFL, CMT, GCC
Sirakaya 2020 [29]	Cross-sectional	Turkey	28.54 ± 4.98	29.26 ± 5.21	72	37	35	Rotterdam	Newly Diagnosed	Heidelberg Spectralis	26.8 ± 4.5	24.05 ± 5.4	GCL
Sumer 2025 [30]	Case-control	Turkey	27 ± 5.40	29.08 ± 4.96	150	120	30	Rotterdam	NR	Heidelberg SD-OCT	25.7 ± 5.31	24.8 ± 4.2	RNFL, CMT, SFCT
Teberik 2017 [31]	Prospective comparative	Turkey	23.4 ± 4.5	24.0 ± 6.1	99	46	53	Rotterdam	NR	Topcon 3D OCT-1000	NR	NR	RNFL
Yener 2021 [32]	Descriptive clinical	Turkey	24.5 ± 4.33	26 ± 5.23	94	59	35	NIH	NR	Optovue RTVue XR Avanti	26.16 ± 5.63	22.54 ± 1.83	RNFL, CMT, GCC, SCP, DCP, FAZ

Risk-of-Bias Assessment 

All 15 observational studies were assessed using the NOS for quality assessment, and all but one study scored at least 7, indicating overall good methodological quality; a single case-control study scored 6, reflecting fair quality (Tables 2, 3). In addition, we used the GRADE framework to evaluate the certainty of the body of evidence for the primary outcomes: RNFL thickness, CMT, SFCT, SCP VD, and DCP VD. According to five downgrading factors: risk of bias, inconsistency, indirectness, imprecision, and publication bias. A summary of pooled effect estimates and certainty ratings based on the GRADE assessment is presented in the Summary of Findings table (Table 4). The results suggested moderate certainty in the evidence for RNFL thickness and CMT. The certainty of evidence for SCP VD and DCP VD was low, while SFCT was graded as very low. The detailed assessment results of evidence quality (Table 5). 

**Table 2:** Risk-of-bias assessment for the cross-sectional studies with Newcastle-Ottawa Scale (NOS)

Cross-sectional studies	Selection (maximum of 4 stars)				Comparability (maximum of 2 stars)	Outcome (maximum of 3 stars)			Total score (out of 9)
	Representativeness of the exposed cohort	Selection of the non- exposed cohort	Ascertainment of exposure	Demonstration that the outcome of interest was not present at the start of the study	Comparability of cohorts on the basis of design or analysis	Assessment of the outcome	Was the follow-up long enough for the outcome to occur	Adequacy of the follow-up of cohorts	
										Acar 2021 [11]	*	*	*		*	*	*	*	7
Acmaz 2014 [10]	*	*	*		*	*	*	*	7
Adiyeke 2017 [21]	*	*	*	*	*	*		*	7
Alpogan 2023 [22]	*	*	*	*	**	*	*	*	9
Demir 2013 [23]	*	*	*	*	*	*		*	7
Elbeyli 2021 [12]	*	*	*	*	**	*		*	8
Gunes 2025 [24]	*	*	*	*	**	*		*	8
Junior 2015 [26]	*	*	*	*	**	*	*	*	9
Puthiyedath 2022 [27]	*	*	*	*	*	*	*	*	8
Shiromani 2022 [28]	*	*	*		**	*		*	7
Sirakaya 2020 [29]	*	*	*	*	**	*		*	8
Teberik 2017 [31]	*	*	*	*	**	*		*	8
Yener 2021 [32]	*	*	*	*	**	*	*	*	9

**Table 3:** Risk-of-bias assessment for the case-control studies with Newcastle-Ottawa Scale (NOS)

Case-control	Selection (Maximum 4 stars)				Comparability (Maximum 2 stars)	Outcome (Maximum 3 stars)			Total Score (out of 9)
	Is the case definition adequate?	Representativeness of the cases	Selection of controls	Definition of controls	Comparability of cohorts in the basis of the design or analysis	Ascertainment of exposure	Same method of ascertainment for cases and controls	Non-response rate	
										Sumer 2025 [30]	*		*	*		*	*	*	6
Icoz 2026 [25]	*		*	*	**	*	*	*	8

**Table 4:** Summary of findings

Outcome	No. of studies (no. of participants)	Mean difference (95% CI)	Certainty of evidence (GRADE)	Comments
Average RNFL thickness (global peripapillary)	11 studies (1,007)	+3.75 µm (+2.48 to +5.01)	Moderate	Significant thickening. Evidence consistently shows RNFL thickening in PCOS. Heterogeneity was low (I^2^ = 22%).
Central macular thickness (CMT)	9 studies (810)	-3.29 µm (-6.46 to -0.11)	Moderate	Marginal thinning. Statistically significant reduction, but the confidence interval nearly touches zero, suggesting the clinical impact may be negligible.
Subfoveal choroidal thickness (SFCT)	6 studies (640)	Unspecified disease duration: +89.01 µm (86.44 to 91.59) Newly Diagnosed: +24.91 µm (-12.76 to 62.58)	Very Low	Dependent on disease duration. High heterogeneity (I^2^ = 99%) precludes a single average. Sensitivity analysis shows thickening in the unspecified disease duration cases, but there is no difference in newly diagnosed patients.
Superficial vessel density (SCP) (whole image)	3 studies (236)	-0.13% (-0.93 to +0.68)	Low	No difference. Vascular density appears preserved. Confidence intervals are wide, crossing the line of no effect.
Deep vessel density (DCP) (whole image)	3 studies (236)	+0.34% (-1.13 to +1.82)	Low	No difference. Similar to superficial layers, no evidence of microvascular dropout was found in the deep plexus.

**Table 5:** Evaluation of the GRADE evidence quality a. Absence of allocation concealment and blinding (inherent to cross-sectional/observational study design). b. The p-value of the heterogeneity test was <0.1, and I² > 50%. c. The 95% confidence interval crosses the line of no effect, and the sample size is relatively small. RNFL: retinal nerve fiber layer; CMT: central macular thickness; SFCT: subfoveal choroidal thickness; SCP VD: superficial capillary plexus vascular density; DCP VD: deep capillary plexus vascular density; MD: mean difference; GRADE: Grading of Recommendations Assessment, Development and Evaluation

Outcome	MD, 95% CI	Risk of bias	Inconsistency	Indirectness	Imprecision	Publication bias	Upgrade quality	Quality
RNFL thickness	3.75 (2.48 to 5.01)	Serious^a^	No	No	No	Undetected	None	Moderate
CMT	-3.29 (-6.46 to -0.11)	Serious^a^	No	No	No	Undetected	None	Moderate
SFCT	58.03 (15.37 to 100.68)	Serious^a^	Very serious^b^	No	No	Undetected	None	Very Low
SCP VD	-0.13 (-0.93 to 0.68)	Serious^a^	No	No	Serious^c^	Undetected	None	Low
DCP VD	0.34 (-1.13 to 1.82)	Serious^a^	No	No	Serious^c^	Undetected	None	Low

Primary Outcomes 

**Figure 2:**
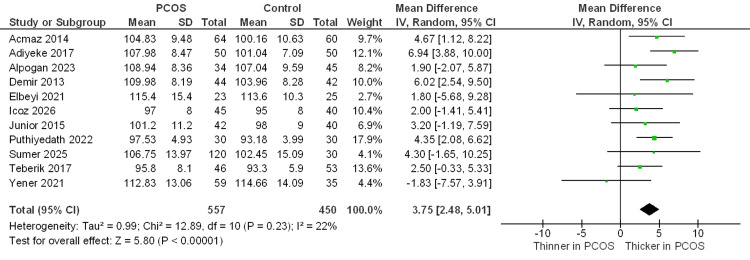
Forest plot of the average RNFL thickness between the PCOS and control groups Studies included: [10,12,21-23,25-27,30-32]

Average RNFL thickness µm: A total of 11 studies reported average peripapillary retinal nerve fiber layer (RNFL) thickness. Meta-analysis revealed that the PCOS group had significantly thicker global RNFL compared with healthy controls (MD = +3.75 µm; 95% CI 2.48 to 5.01; P < 0.00001). Heterogeneity among the included studies was low (I² = 22%) (Figure 2). 

**Figure 3:**
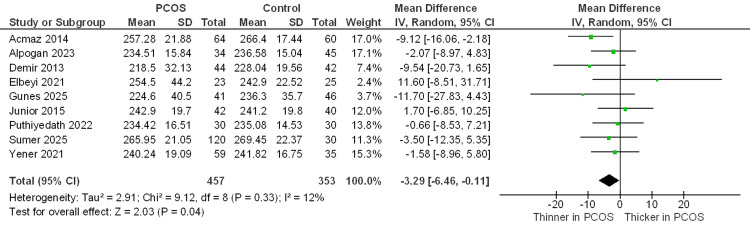
Forest plot of the central macular thickness between the PCOS and control groups Studies included: [10,12,22–24,26,27,30,32]

CMT µm: A total of nine studies measured the CMT. Pooled analysis showed that the PCOS group had a modest but statistically significant reduction in CMT compared with controls (MD = −3.29 µm; 95% CI −6.46 to −0.11; P = 0.04). Between-study heterogeneity was low (I² = 12%) (Figure 3). 

**Figure 4:**
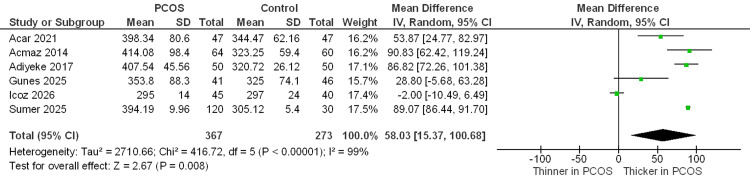
Forest plot of the subfoveal choroidal thickness between the PCOS and control groups Studies included: [10,11,21,24,25,30]

SFCT µm: Six studies assessed the SFCT. Meta-analysis demonstrated significantly increased SFCT in women with PCOS compared with controls (MD = +58.03 µm; 95% CI 15.37 to 100.68; P < 0.00001). Significant heterogeneity was observed (I² = 99%) (Figure 4).

**Figure 5:**
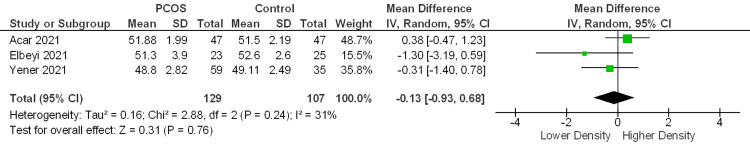
Forest plot of the whole superficial capillary plexus (SCP) vessel density between the PCOS and control groups Studies included: [11,12,32]

**Figure 6:**
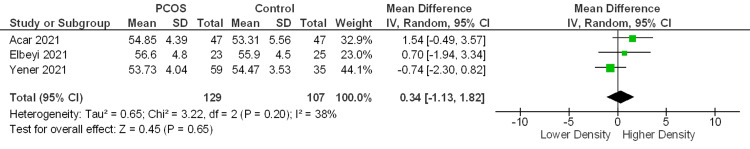
Forest plot of the whole deep capillary plexus (DCP) vessel density between the PCOS and control groups Studies included: [11,12,32]

SCP and DCP vessel density %: The vessel density measurements of the whole SCP were obtained from three studies. The pooled analysis indicated no statistically significant difference between the PCOS and control groups (MD = −0.13%; 95% CI −0.93 to 0.68; P = 0.76), displaying low-to-moderate heterogeneity (I² = 31%) (Figure 5). Likewise, the same three studies assessed the whole DCP vessel density. Meta-analysis revealed no significant difference in DCP density among groups (MD = +0.34%; 95% CI −1.13 to 1.82; P = 0.65), showing moderate heterogeneity (I² = 38%) (Figure 6).

Secondary Outcomes 

Exploratory and sensitivity analyses of additional structural and microvascular parameters are presented in Appendices C-E. Consistent with the primary OCTA outcomes, no significant differences were observed in foveal avascular zone (FAZ) area or radial peripapillary capillary (RPC) density between PCOS patients and controls (P > 0.05 for all comparisons) (Appendix C).

By contrast, additional structural analyses showed a localized pattern of retinal thickening. The average macular ganglion cell complex (GCC) thickness was significantly increased in the PCOS group (MD = +2.16 µm; 95% CI: 0.85 to 3.47; P = 0.001) (Appendix D), primarily in the inferior sector, where a specific thickening (MD = +2.19 µm; 95% CI: 0.50 to 3.87; P = 0.01; I² = 0%) (Appendix D). Topographic analysis of peripapillary RNFL thickness demonstrated modest nasal quadrant thickening, while superior, inferior, and temporal quadrants showed no significant differences (Appendix D). Similarly, within the macular retina, parafoveal thickness did not differ across quadrants, whereas the perifoveal analysis identified significant thickening limited to the nasal quadrant (MD = +12.19 µm; P = 0.04) (Appendix D), with other sectors unchanged. 

Sensitivity Analyses 

Leave-one-out sensitivity analyses were conducted for nasal and superior peripapillary RNFL thickness (Appendix E) and for parafoveal macular thickness (Appendix E), demonstrating the stability of pooled effect estimates following sequential study exclusion. In addition, device-stratified subgroup analyses for the perifoveal nasal and temporal sectors (Appendix E) reduced heterogeneity without altering the direction of the pooled effects. Analysis of SFCT showed high heterogeneity (I² = 99%). Subgroup analysis based on disease duration revealed that the unspecified disease duration group showed a significant and consistent increase in choroidal thickness (MD = 89.01 μm; 95% CI: 86.44 to 91.59; I² = 0%; P < 0.00001). While the newly diagnosed group showed high heterogeneity and no statistically significant difference compared to controls (MD = 24.91 μm; 95% CI: −12.76 to 62.58; I² = 87%; P = 0.19), the test for subgroup differences was significant (P = 0.0009) (Appendix E). Furthermore, sensitivity analysis excluding the preprint study (Gunes et al. [24]) did not alter the direction or significance of these results. The second outcome from the preprint study was CMT; the initial pooled analysis indicated a significant decrease in CMT (P = 0.04), but exclusion of the preprint resulted in a loss of statistical significance (P = 0.07). Finally, funnel plot inspection and Egger’s regression test did not indicate statistically significant effects (t = −1.28, df = 9, P = 0.233) for the primary outcome of RNFL thickness, suggesting a low likelihood of major publication bias (Appendix F).

Discussion

Main Findings

This systematic review and meta-analysis summarized the evidence of retinal, choroidal, and microvascular changes measured by OCT and OCTA in women with PCOS. The primary findings suggest that PCOS is associated with significant thickening in the peripapillary RNFL and SFCT, as well as a modest reduction in CMT, which demonstrated sensitivity to exclusion of non-peer reviewed data. By comparison, microvascular parameters as measured by OCTA, such as the density of superficial and deep capillary plexus, as well as the area in the FAZ and the density of RPC, showed no significant differences between PCOS patients and controls. Together, the results indicate that structural retinal and choroidal remodeling can take place in PCOS without the microvascular rarefaction, which indicates a lack of association between tissue architecture and capillary density at an initial disease spectrum. All observed associations should be interpreted as non-causal, as the included studies were observational in design (predominantly cross-sectional with two case-control) and do not permit temporal or mechanistic inference. 

Interpretation of Structural Findings

The increase in global peripapillary RNFL thickness represents one of the most robust findings of this meta-analysis, which is substantiated by a low degree of between-study heterogeneity and a tangible consistency in the direction of the effect across OCT platforms. The RNFL thickening has been reported in a number of systemic, metabolic, and neuroinflammatory diseases and is usually considered an expression of subclinical axonal swelling, impaired axoplasmic transport, or low-grade inflammatory edema rather than true axonal proliferation or neuroprotection [33-35]. In that regard, the RNFL emerges as especially susceptible to metabolic and hormonal derangements of systemic metabolism that are defining PCOS. 

Notably, there was a statistically significant but small decrease in CMT that was observed to accompany RNFL thickening. Even though the magnitude of CMT thinning was low, it was found to be low in heterogeneity and highly directional, which suggests internal consistency across studies, although the stability of this association appears sensitive to study inclusion. However, this observation warrants cautious interpretation, as sensitivity analysis excluding non-peer-reviewed data rendered the association non-significant (P = 0.07), suggesting that macular thinning may be a subtle trend rather than a definitive clinical feature.

Central macular thinning has been described in the early neurodegenerative and metabolic disorders and could be evidence of early neuronal or synaptic loss in the foveal region [36]. Anatomically, the fovea is avascular and morphologically specialized, with limited structural reserve to accommodate subtle tissue perturbations, potentially rendering it more sensitive to early neurodegenerative processes [37,38]. 

These findings of RNFL thickening and CMT thinning coexisting suggest that there is no consistent retinal hypertrophic response in PCOS but rather a disproportionate distribution of retinal involvement. This dissociation in space can imply that various retinal segments may have different responses to systemic metabolic, inflammatory, or hormonal stresses, with some areas showing edema or axonal swelling and others showing early thinning. 

Topographically selective patterns of retinal involvement were found through secondary and exploratory analyses. Such results were predominant thickening of the nasal peripapillary RNFL, inferior sector-predominant GCC thickening, and nasal perifoveal macular thickening. Though not pre-specified as primary outcomes and to be approached with caution, these results are reproducible across analyses, which means that retinal involvement in PCOS may be regionally selective rather than diffuse. 

Anatomically, the nasal retina is where the papillomacular bundle is densely concentrated, and it is located in close relation to the optic nerve head, which makes it possibly more sensitive to subtle changes in axonal transport, interstitial fluid dynamics, or inflammatory signaling [39,40] The consistent involvement of the nasal region across both peripapillary and macular assessments further supports the possibility of region-specific vulnerability within retinal architecture. 

Interpretation of Vascular Findings

Although there is evident structural alteration on OCT, OCTA studies have shown that there is preserved microvascular density on all parameters assessed, such as superficial and deep capillary plexus, FAZ area, and RPC density. This result contrasts with ischemic retinopathies, including diabetes mellitus, where microvascular dropout is common and may precede or even parallel with structural injury, and suggests that PCOS-related ocular involvement may initially be driven by non-ischemic mechanisms [41].

This coexistence of structural changes with maintained capillary density suggests that hormonal, metabolic, or neuroinflammatory pathways may have primary effects on retinal tissue architecture; it may happen before the occurrence of irreversible microvascular injury. Alternatively, there might be some undetected endothelial dysfunction or capillary flow changes that fall below the current OCTA technology detection limit, particularly given the cross-sectional nature of the data and the lack of standardized reporting for disease duration across studies. 

Heterogeneity and Disease Duration Effects

Although the SFCT was higher in the pooled results, interpretation was challenging due to the extremely high heterogeneity. Given the I² of 99% and very low GRADE certainty, the pooled SFCT estimate should be interpreted as hypothesis-generating rather than definitive. When the data were sorted by length of disease, the newly diagnosed group did not exhibit any notable change, yet remained highly heterogeneous, while the group with an unknown duration showed distinct and continuous thickening. These results imply that choroidal remodeling may represent a progressive structural adaptation to cumulative metabolic dysfunction rather than occurring immediately following a PCOS diagnosis. 

The choroidal tissue is highly vascular and sensitive to hormonal changes; an increase in thickness is derived from endothelial function, inflammatory mediators, and systemic metabolic status [6,38]. In PCOS, established systemic metabolic dysregulation contributes to vascular dilation and increased choroidal blood volume, which manifests as an increase in the SFCT. The absence of consistent thickening in the newly diagnosed cohort implies that early-stage disease may lack the cumulative 'dose' of metabolic insult required to drive measurable structural change, or that incipient remodeling is too subtle to be captured by current protocols. 

It is important to note that the lack of explicit disease duration reporting in the 'unspecified' group precludes formal time-course analysis. Consequently, observations regarding disease chronicity should be interpreted as hypothesis-generating. However, the marked reduction of heterogeneity within the established disease cohort provides convergent evidence that disease duration and cumulative metabolic burden are likely key modulators of choroidal involvement in PCOS.

Literature Context

Previous qualitative and integrative reviews have summarized reported retinal and choroidal changes in PCOS but have not imposed quantitative pooling due to high heterogeneity. The current systematic review and meta-analysis advance this body of literature by combining the existing data on OCT and OCTA while contextualizing variability through supplementary sensitivity and subgroup analyses. Methodological differences in search strategy may partly explain discrepancies between reviews. In contrast with the previous integrative reviews that made use of language and date constraints, this analysis had no language or date limits, thus being eligible for both older and non-English studies. Although this wider focus ensures more completeness, it may also increase heterogeneity, emphasizing the need for cautious interpretation and robust sensitivity analyses when evaluating ocular imaging findings in PCOS. 

Clinical Implications

These findings are not in favor of regular ophthalmic screening of asymptomatic PCOS women. Nevertheless, the monitored structural changes using OCT raise the possibility that retinal and choroidal imaging can be used as non-invasive research methods to investigate systemic neuro-metabolic and inflammatory mechanisms linked to PCOS. The lack of association between the structural changes and maintained microvascular density also indicates the possibility of a window period when the early investigation can be done before the damage to the vascular structures is irreversible. 

The longitudinal designs should be put as a priority in future studies to establish whether RNFL thickening is merely a temporary process before axonal thinning occurs with the progression of the disease or aging. Standardization of OCT and OCTA acquisition protocols should be implemented, which includes the control for diurnal variation and the incorporation of detailed metabolic phenotyping. Accounting for these variables will clarify the mechanisms of this disease. 

Limitations

Several limitations were encountered, most of the studies included were based in one geographic area, which limits generalizability. Measurement heterogeneity could have been caused by variability in OCT platforms and segmentation algorithms. Additionally, one included study was a preprint at the time of analysis, and although sensitivity analyses were performed, the absence of peer review may introduce residual uncertainty regarding effect stability. The effects of time of day on the findings of OCTA studies were not considered, even though a diurnal change in choroidal and vascular parameters was known. Body mass index reporting was not done consistently, and the lack of stratified weight cohorts limited our ability to assess obesity-independent effects. Disease duration was poorly characterized across studies, hindering the formal stage-based analyses. Lastly, cross-sectional observational designs constrain the ability to make causal inferences and determine the course of development with time.

## Conclusions

Overall, the present systematic review and meta-analysis indicate consistent evidence of retinal and choroidal structural changes linked to PCOS, namely, consistent thickening of the peripapillary retinal nerve fiber layer and the evidence suggestive of thinning of the central macula, as well as the enlargement of the subfoveal choroid that is present in the absence of microvascular density change on OCTA. These results highlight that the eye can serve as a window to early systemic pathophysiology in PCOS and that structural remodeling can pre-empt overt microvascular damage, with disease stage modulating the magnitude and consistency of these changes. 

## Figures and Tables

**Figure 7:**
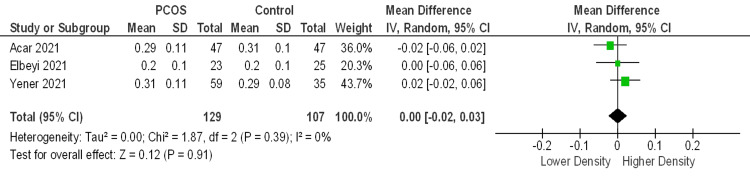
Forest plot comparing foveal avascular zone (FAZ) area between PCOS patients and controls.

**Figure 8:**
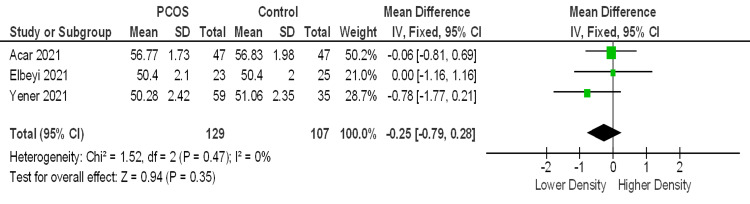
Forest plot comparing radial peripapillary capillary (RPC) vessel density between PCOS patients and controls.

**Figure 9:**
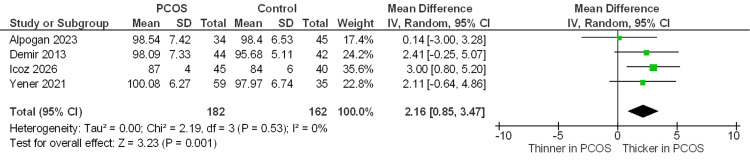
Forest plot of the average macular ganglion cell complex (GCC) thickness in PCOS patients versus controls.

**Figure 10:**
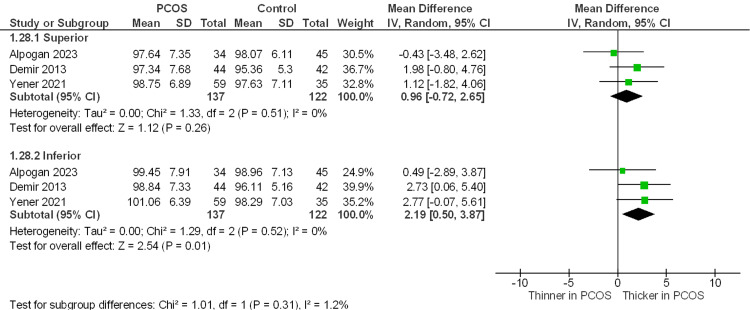
Forest plot of the macular ganglion cell complex (GCC) thickness stratified by the superior and inferior hemispheres.

**Figure 11:**
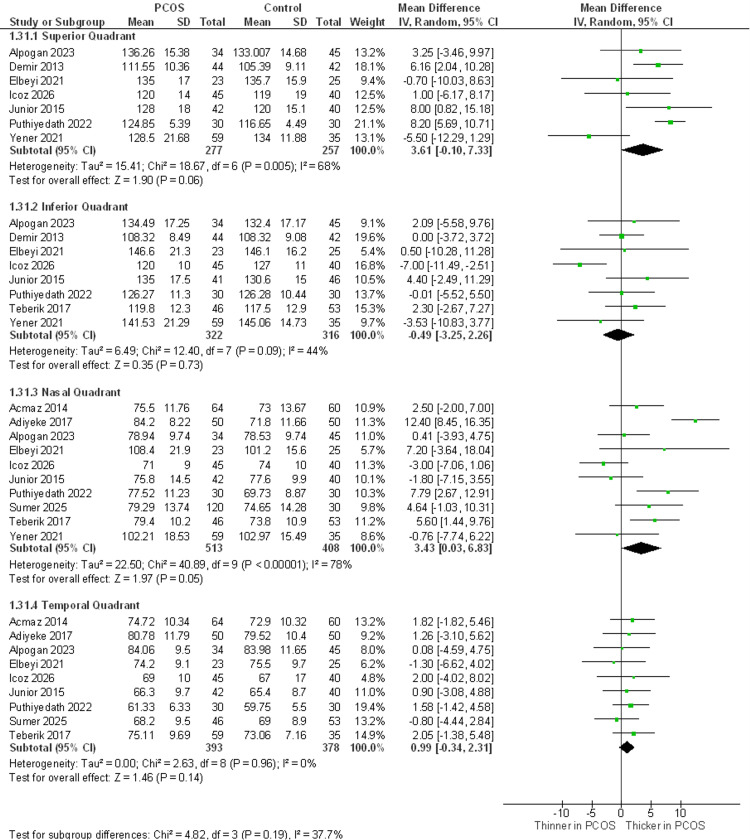
Forest plot of peripapillary RNFL thickness stratified by quadrant (superior, inferior, nasal, temporal).

**Figure 12:**
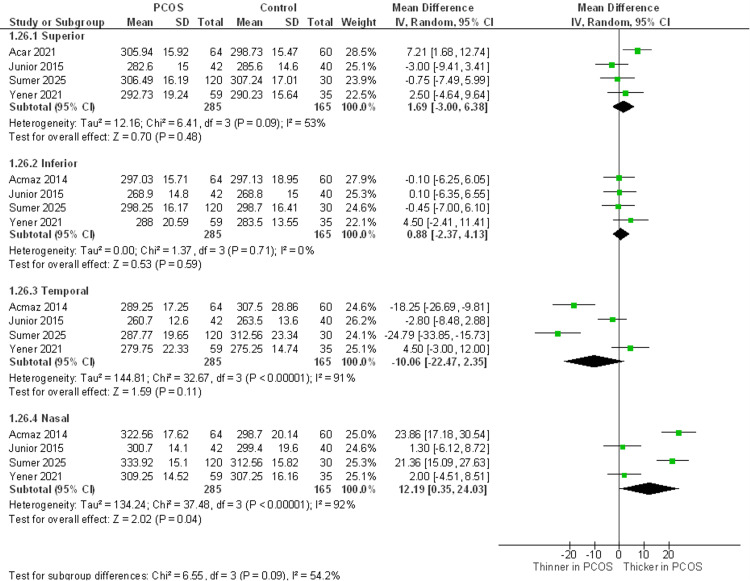
Forest plot of the perifoveal macular thickness stratified by quadrant (superior, inferior, nasal, and temporal).

**Figure 13:**
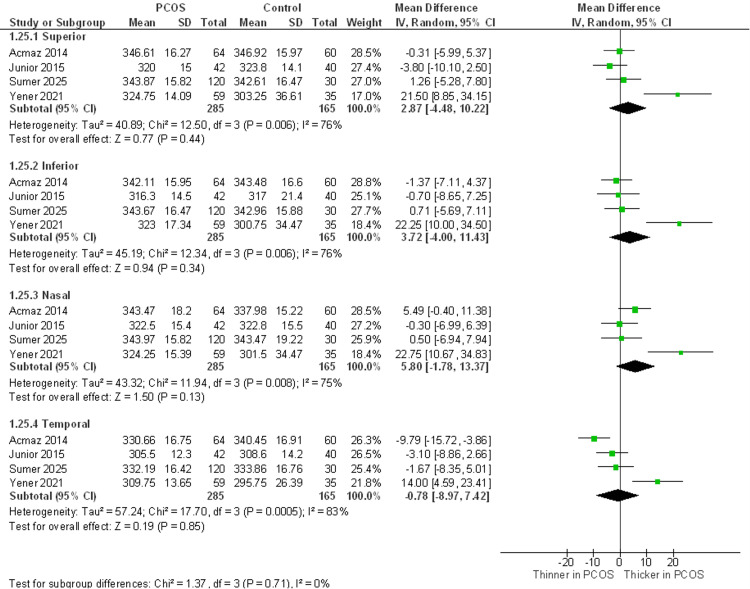
Forest plot of the parafoveal macular thickness stratified by quadrant (superior, inferior, nasal, and temporal).

**Figure 14:**
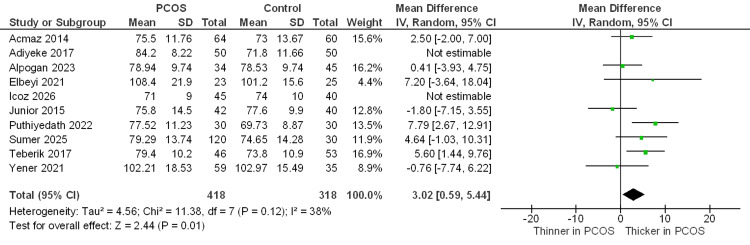
Leave-one-out sensitivity analysis of nasal peripapillary retinal nerve fiber layer (RNFL) thickness.

**Figure 15:**
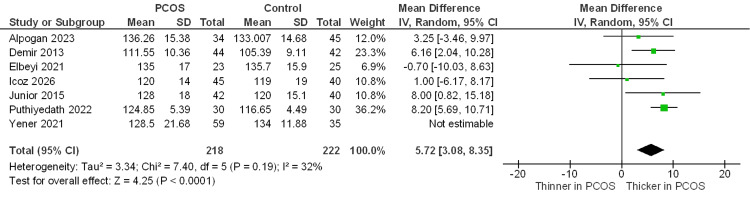
Forest plot of the superior sector retinal thickness comparing women with polycystic ovary syndrome (PCOS) and healthy controls.

**Figure 16:**
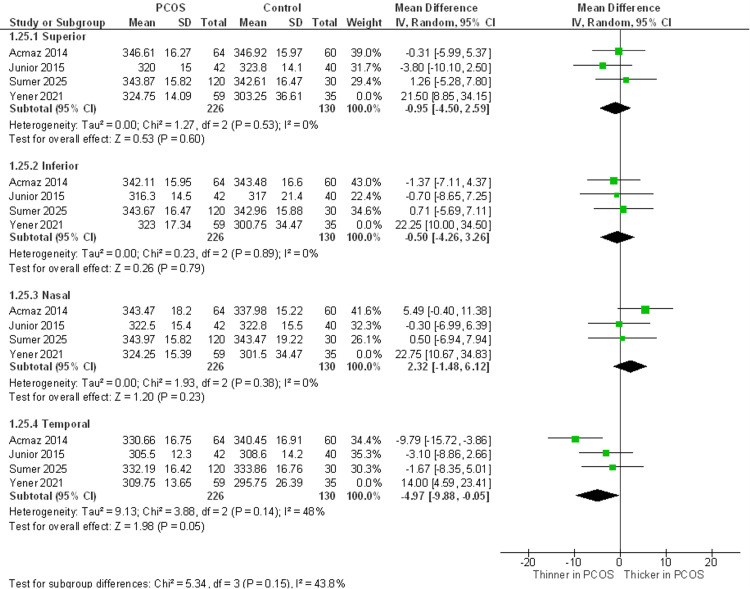
Leave-one-out sensitivity analysis of the parafoveal macular thickness by quadrant.

**Figure 17:**
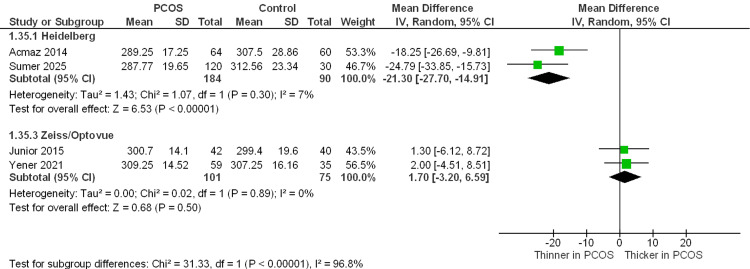
Forest plot of the nasal perifoveal macular thickness comparing PCOS patients and controls, stratified by the OCT device manufacturer.

**Figure 18:**
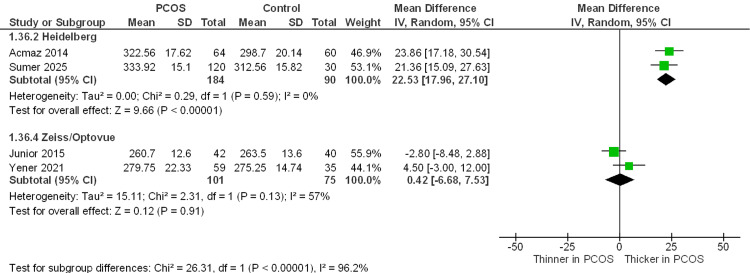
Forest plot of the temporal perifoveal macular thickness comparing PCOS patients and controls, stratified by the OCT device manufacturer.

**Figure 19:**
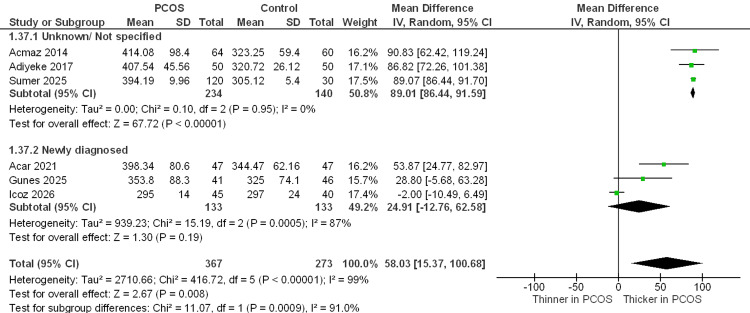
Subgroup analysis of the subfoveal choroidal thickness (SFCT) stratified by study population (newly diagnosed vs. unspecified duration).

**Figure 20:**
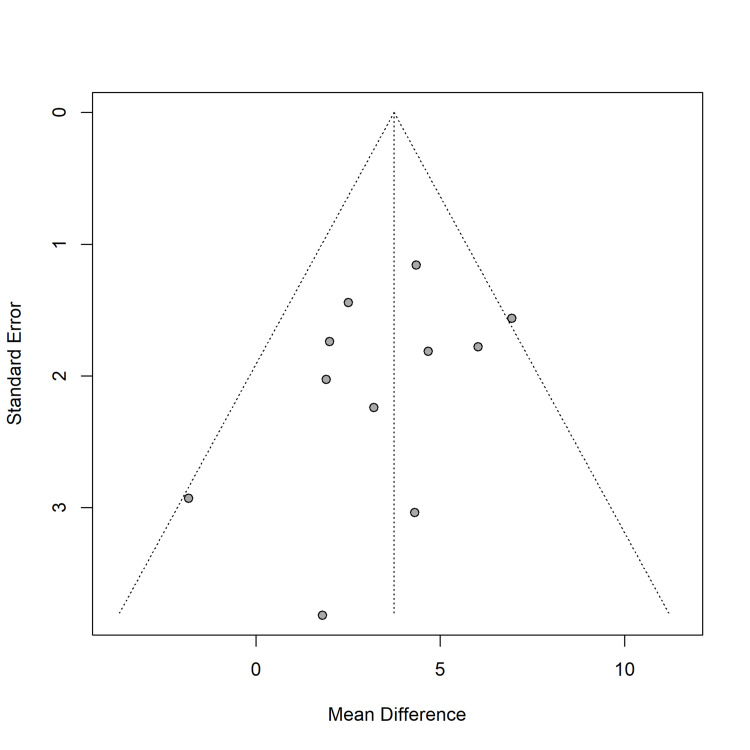
Funnel plot for the average retinal nerve fiber layer (RNFL) thickness, showing no significant small-study effects (Egger’s test P = 0.233).

## Appendices

Appendix A: Search strategy

Pubmed

("Polycystic Ovary Syndrome"[MeSH Terms] OR "polycystic ovary syndrome"[Title/Abstract] OR "polycystic ovarian syndrome"[Title/Abstract] OR "PCOS"[Title/Abstract] OR "Stein-Leventhal syndrome"[Title/Abstract] OR "Stein Leventhal syndrome"[Title/Abstract] OR polycystic ovar*[Title/Abstract] OR sclerocystic ovar*[Title/Abstract]) 

AND 

 ("Tomography, Optical Coherence"[MeSH Terms] OR "optical coherence tomography"[Title/Abstract] OR optical coherence tomograph*[Title/Abstract] OR "OCT"[Title/Abstract] OR "OCTA"[Title/Abstract] OR "OCT-A"[Title/Abstract] OR "OCT angiography"[Title/Abstract] OR "optical coherence tomography angiography"[Title/Abstract] OR "spectral domain OCT"[Title/Abstract] OR "SD-OCT"[Title/Abstract] OR "swept source OCT"[Title/Abstract] OR "SS-OCT"[Title/Abstract] OR "enhanced depth imaging"[Title/Abstract] OR "EDI-OCT"[Title/Abstract])

Embase

('polycystic ovary syndrome'/exp OR 'polycystic ovary syndrome':ti,ab,kw OR 'polycystic ovarian syndrome':ti,ab,kw OR 'pcos':ti,ab,kw OR 'stein-leventhal syndrome':ti,ab,kw OR 'stein Leventhal syndrome':ti,ab,kw OR (polycystic NEAR/0 ovar*):ti,ab,kw OR (sclerocystic NEAR/0 ovar*):ti,ab,kw)

AND

 ('optical coherence tomography'/exp OR 'optical coherence tomography angiography'/exp OR'optical coherence tomography':ti,ab,kw OR 'optical coherence tomograph*':ti,ab,kw OR'oct':ti,ab,kw OR 'octa':ti,ab,kw OR 'oct-a':ti,ab,kw OR 'oct angiography':ti,ab,kw OR 'opticalcoherence tomography angiography':ti,ab,kw OR 'spectral domain oct':ti,ab,kw OR 'sd-oct':ti,ab,kw OR 'swept source oct':ti,ab,kw OR 'ss-oct':ti,ab,kw OR 'enhanced depth imaging':ti,ab,kw OR 'edi-oct':ti,ab,kw)

Line-by-line 

#1 'polycystic ovary syndrome'/exp

#2 'polycystic ovary syndrome':ti,ab,kw OR 'polycystic ovarian syndrome':ti,ab,kw OR

'pcos':ti,ab,kw OR 'stein-leventhal syndrome':ti,ab,kw OR 'stein leventhal syndrome':ti,ab,kw OR

(polycystic NEAR/0 ovar*):ti,ab,kw OR (sclerocystic NEAR/0 ovar*):ti,ab,kw

#3    #1 OR #2

#4 'optical coherence tomography'/exp

#5 'optical coherence tomography angiography'/exp

#6 'optical coherence tomography':ti,ab,kw OR 'optical coherence tomograph*':ti,ab,kw OR

'oct':ti,ab,kw OR 'octa':ti,ab,kw OR 'oct-a':ti,ab,kw OR 'oct angiography':ti,ab,kw OR 'optical

coherence tomography angiography':ti,ab,kw OR 'spectral domain oct':ti,ab,kw OR 'sd-oct':ti,ab,kw OR 'swept source oct':ti,ab,kw OR 'ss-oct':ti,ab,kw OR 'enhanced depth imaging':ti,ab,kw OR 'edi- oct':ti,ab,kw

#7        #4 OR #5 OR #6

#8        #3 AND #7

Scopus

TITLE-ABS-KEY("polycystic ovary syndrome" OR "polycystic ovarian syndrome" OR "PCOS" OR "Stein- Leventhal syndrome" OR "Stein Leventhal syndrome" OR polycystic W/0 ovar* OR sclerocystic W/0 ovar*)

AND

TITLE-ABS-KEY("optical coherence tomography" OR "optical coherence tomograph*" OR "OCT" OR "OCTA" OR "OCT-A" OR "OCT angiography" OR "optical coherence tomography angiography" OR "spectral domain OCT" OR "SD-OCT" OR "swept source OCT" OR "SS-OCT" OR "enhanced depth imaging" OR "EDI-OCT")

WoS

TS=("polycystic ovary syndrome" OR "polycystic ovarian syndrome" OR "PCOS" OR "Stein-Leventhal syndrome" OR "Stein Leventhal syndrome" OR polycystic NEAR/0 ovar* OR sclerocystic NEAR/0 ovar*)

AND

TS=("optical coherence tomography" OR "optical coherence tomograph*" OR "OCT" OR "OCTA" OR "OCT-A" OR "OCT angiography" OR "optical coherence tomography angiography" OR "spectral domain OCT" OR "SD-OCT" OR "swept source OCT" OR "SS-OCT" OR "enhanced depth imaging" OR "EDI-OCT")

Cochrane CENTRAL

#1 ([mh "Polycystic Ovary Syndrome"] OR "polycystic ovary syndrome":ti,ab,kw OR "polycystic ovarian syndrome":ti,ab,kw OR "PCOS":ti,ab,kw OR "Stein-Leventhal syndrome":ti,ab,kw OR "Stein Leventhal syndrome":ti,ab,kw OR (polycystic NEXT ovar*):ti,ab,kw OR (sclerocystic NEXT ovar*):ti,ab,kw) 

AND

#2 ([mh "Tomography, Optical Coherence"] OR "optical coherence tomography":ti,ab,kw OR (optical NEXT coherence NEXT tomograph*):ti,ab,kw OR "OCT":ti,ab,kw OR "OCTA":ti,ab,kw OR "OCT-A":ti,ab,kw OR "OCT angiography":ti,ab,kw OR "optical coherence tomography angiography":ti,ab,kw OR "spectral domain OCT":ti,ab,kw OR "SD-OCT":ti,ab,kw OR "swept source OCT":ti,ab,kw OR "SS-OCT":ti,ab,kw OR "enhanced depth imaging":ti,ab,kw OR "EDI-OCT":ti,ab,kw)

#1 AND #2

Google Scholar

(PCOS OR "Polycystic Ovary Syndrome") AND ("Optical Coherence Tomography" OR OCT OR OCTA)

Appendix B: Outcome-specific data handling and exclusions

For studies reporting multiple PCOS phenotypes, we pooled the subgroups into a single experimental group to avoid unit-of-analysis errors associated with double-counting the control group. We prioritized data from the “right eye” or “randomly selected eye” when reported. If studies reported bilateral averages without adjustment for inter-eye correlation, the sample size was treated as the number of patients rather than eyes to maintain statistical conservatism.

Outcome-specific exclusions were applied where anatomical or methodological incompatibilities precluded valid quantitative synthesis. Eligible data from these studies were retained for other outcomes where such limitations were not present.

Shiromani et al. (2022): This study was excluded from quantitative meta-analysis because it reported considerable implausible thickness measurements of the retinal nerve fiber layer. Isolated retinal nerve fiber layer thickness is typically around 100 µm, while the measurement values included in this study exceeded the physiological limits (>220 µm). These values suggest that the study measured total retinal thickness; therefore, the data could not be pooled with the remaining studies.

Sirakaya et al. (2020): This study was excluded from quantitative meta-analysis because it did not measure peripapillary retinal nerve fiber layer analyses; they obtained measurements at the center of the fovea (macular retinal nerve fiber layer). As for the other outcomes reported in this study, they did not meet the prespecified outcomes of interest.

Teberik et al. (2017): This study was excluded only from the superior quadrant retinal nerve fiber layer analysis because the control group showed implausibly high variance. We contacted the authors, and updated values were provided; for example, correction of the reported standard deviation from 138.2 µm to 38.2 µm. However, the revised values remained more than threefold higher than the pooled cohort average (~12 µm), suggesting persistent sectoral segmentation error. This study was retained for the remaining quadrants, where variance was statistically consistent with the other included studies.

This selective, outcome-specific inclusion strategy ensured anatomical validity and statistical robustness while maximizing the use of available data.

Appendix C: Additional secondary OCTA outcomes

Foveal Avascular Zone (FAZ) Area and Radial Peripapillary Capillary (RPC) Density

In secondary analyses, we examined OCT angiography-derived microvascular parameters and found no statistically significant differences between women with PCOS and healthy controls. Specifically, pooled analyses showed no difference in foveal avascular zone (FAZ) area (MD = 0.00 mm²; P = 0.91) and no significant difference in radial peripapillary capillary (RPC) vessel density (MD = −0.25%; P = 0.35) (Figures 7, 8).

Appendix D: Additional secondary OCT outcomes

Ganglion Cell Complex (GCC) Thickness

A meta-analysis of global GCC displayed that the thickness in the PCOS group was higher compared to the normal control (MD = +2.16 µm; 95% CI 0.85 to 3.47; P = 0.001), with no observed heterogeneity (I² = 0%) (Figure 9). We proceeded with regional Subgroup analysis, and it showed a statistically significant increase in the inferior sector (MD = +2.19 µm; P = 0.01), whereas no significant difference was observed in the superior sector (P = 0.26) (Figure 10).

Topographic Peripapillary RNFL Thickness

We created a topographic peripapillary RNFL thickness subgroup analysis. There was no significant difference between women with PCOS and the control group in the inferior and temporal quadrants; however, the nasal quadrant showed thicker RNFL in the PCOS group (MD = +3.43 µm; 95% CI 0.03 to 6.83; P = 0.05). Substantial heterogeneity was observed in the nasal quadrant analysis (I² = 78%) (Figure 11).

Perifoveal and Parafoveal Macular Thickness

We created subgroup analysis for topographic analysis of perifoveal macular thickness, which demonstrated a statistically significant increase in the nasal quadrant (MD = +12.19 µm; 95% CI 0.35 to 24.03; P = 0.04), with substantial heterogeneity (I² = 92%), while no significant differences were observed in the remaining quadrants (Figure 12). We repeated the approach for parafoveal macular thickness, and it showed no statistically significant differences across any the four quadrants (Figure 13).

Appendix E: Sensitivity analyses

Nasal Peripapillary RNFL Thickness

The Initial analysis for nasal peripapillary RNFL thickness demonstrated considerable heterogeneity (I² = 78%). We performed leave-one-out sensitivity analysis and identified two studies contributing to between-study variability. First, Adiyeke et al. reported a larger effect estimate (+12.4 µm), excluding this reduced heterogeneity to 57%. Subsequent exclusion of Icoz et al., which enrolled a newly diagnosed PCOS cohort, further reduced heterogeneity to 38% (Figure 14). In all sensitivity models, the pooled effect remained statistically significant.

Superior Peripapillary RNFL Quadrant

Initial analysis of the superior peripapillary RNFL quadrant yielded high heterogeneity (I² = 78%). We performed leave-one-out sensitivity analysis and identified Yener et al. (2021) as the major contributor to between-study variability due to a directional inconsistency relative to other included studies. While most studies reported increased thickness in the superior sector in women with PCOS, Yener et al. reported a reduction. Exclusion of Yener et al. reduced heterogeneity to 32% (P = 0.19) (Figure 15).

Parafoveal Macular Thickness

The initial analysis of the parafoveal macula showed no statistically significant differences between women with PCOS and controls across any quadrant (superior P = 0.60; inferior P = 0.79; nasal P = 0.23; temporal P = 0.05). The baseline heterogeneity was high across four quadrants (I² > 75%). Leave-one-out sensitivity analysis identified Yener et al. as a contributor to heterogeneity. Exclusion of this study reduced heterogeneity to 0% in the superior, inferior, and nasal quadrants, while pooled estimates remained non-significant (Figure 16).

Perifoveal Macular Thickness

Initial analysis of perifoveal macular thickness demonstrated no significant differences between PCOS patients and controls in the superior, inferior, or temporal quadrants (P > 0.05 for all comparisons). The nasal perifoveal quadrant showed an increase in thickness in women with PCOS compared with controls (MD = +12.19 µm; P = 0.04), with high heterogeneity in the nasal sector (I² = 92%). We made two subgroups; stratification relied on the devices used. The sensitivity analyses showed a reduction of heterogeneity (I² = 0-7%), while effect estimates varied across OCT platforms; temporal quadrant estimates remained non-significant (Figures 17, 18).

Subfoveal Choroidal Thickness

Subgroup analysis of SFCT stratified by diagnostic status (newly diagnosed vs. unspecified duration) revealed that the pooled effect is driven by the unspecified duration cohort (I² = 0%), whereas newly diagnosed cases show no significant choroidal thickening (I² =87%) (Figure 19).

Appendix F: Funnel plot and Egger’s test for RNFL

The distribution of included studies appeared broadly symmetrical around the pooled mean difference, with no clear visual evidence of funnel plot asymmetry. Consistent with this, Egger’s regression test did not demonstrate statistically significant small-study effects (t = −1.28, df = 9, P = 0.233). Overall, these findings suggest a low likelihood of major publication bias influencing the pooled estimate for RNFL thickness (Figure 20).
